# Energy-Dependent Particle Size Distribution Models for Multi-Disc Mill

**DOI:** 10.3390/ma15176067

**Published:** 2022-09-01

**Authors:** Weronika Kruszelnicka, Marek Opielak, Kingsly Ambrose, Saugirdas Pukalskas, Andrzej Tomporowski, Patrycja Walichnowska

**Affiliations:** 1 Department of Machines and Technical Systems, Faculty of Mechanical Engineering, Bydgoszcz University of Science and Technology, Al. Prof. S. Kaliskiego 7, 85-796 Bydgoszcz, Poland; 2 Department of Agricultural & Biological Engineering, Purdue University, 225 S. University St., West Lafayette, IN 47907, USA; 3 Department of Sustainable Transport and Propulsion Sources, Lublin University of Technology, ul. Nadbystrzycka 36, 20-618 Lublin, Poland; 4 Faculty of Transport Engeneering, Vilnius Gediminas Technical University, Saulekio al. 11, LT-10223 Vilnius, Lithuania

**Keywords:** particle size energy, comminution, biomass, Weibull distribution, Rosin–Rammler–Sterling–Bennet distribution, specific comminution energy, corn, rice

## Abstract

Comminution is important in the processing of biological materials, such as cereal grains, wood biomass, and food waste. The most popular biomaterial grinders are hammer and roller mills. However, the grinders with great potential in the processing of biomass are mills that use cutting, e.g., disc mills. When it comes to single-disc and multi-disc grinders, there are not many studies describing the relationships between energy, motion, material, and processing or describing the effect of grinding, meaning the size distribution of a product. The relationship between the energy and size reduction ratio of disc-type grinder designs has also not been sufficiently explored. The purpose of this paper was to develop models for the particle size distribution of the ground product in multi-disc mills depending on the variable process parameters, i.e., disc rotational velocity and, consequently, power consumption, and the relationship between the grinding energy and the shape of graining curves, which would help predict the product size reduction ratio for these machines. The experiment was performed using a five-disc mill, assuming the angular velocity of the grinder discs was variable. Power consumption, product particle size, and specific comminution energy were recorded during the tests. The Rosin–Rammler–Sperling–Bennet (RRSB) distribution curves were established for the ground samples, and the relationships between distribution coefficients and the average angular velocity of grinder discs, power consumption, and specific comminution energy were determined. The tests showed that the specific comminution energy increases as the size reduction ratio increases. It was also demonstrated that the RRSB distribution coefficients could be represented by the functions of angular velocities, power consumption, and specific comminution energy. The developed models will be a source of information for numerical modelling of comminution processes.

## 1. Introduction

Grinding is an important element in the processing of biological materials, such as cereal grains, woody biomass, food waste, etc., and its purpose is to reduce bulk density and storage areas, improve nutrient release, prepare for further processing, etc. [1,2,3,4,5,6]. In food production processes, e.g., flour production or pellet manufacturing processes, the size of the ground particles is important and is determined using particle size distribution curves [7,8,9,10]. For example, the particle size distribution functions can be used to describe the ground materials, build proper models for grinding processes, and optimise the mill design. There are several known distribution models in the literature that successfully describe particle size distributions, e.g., the Gates–Gaudin–Schuhmann (GGS) distribution, the Rosin–Rammler–Sperling–Bennett (RRSB) distribution, the Gaudin–Meloy distribution, and the Kolmogorov and Gaussian distribution [11,12,13]. In order to determine them, it is required to carry out experimental tests, e.g., sieve analysis, and adjust the theoretical models to set the appropriate characteristic parameters, such as the scale and shape of these distributions [11,12]. The tests conducted for ball mills [14], cylindrical mills [15,16], and hammer mills [1,17] showed that the particle size distribution curves vary depending on the process and design parameters of the mills used for grinding, e.g., the size of the grinding gap, rotational velocities, or the number of balls in ball mills. Furthermore, there is ongoing research to identify the relationship between the particle size of the ground product and the specific comminution energy for mills [18,19,20,21], which in a broader perspective should allow us to understand the comminution process to reduce the energy consumption and to optimise grinding machine designs.

In the case of comminution of biomaterials, e.g., cereal grains, the most popular group of grinders are hammer mills [1,22] and roller mills [23,24,25,26]. The grinders with great potential in biomass processing are the mills that use a cutting action for easy breakage of the fibrous structure [1]. This type of machine includes disc mills, which are generally highly efficient [27,28,29,30]. However, there are not many studies describing the relationships between energy, motion, material, and processing or describing the effect of grinding on the size distribution of product for the disc grinders. The relationship between energy and the size reduction ratio for this type of grinder design has also not been sufficiently explored. Therefore, there is a need for research that would expand the current knowledge and methods by addressing the relationship between the grinding energy and particle size for multi-disc, multi-hole mills. When it comes to multi-disc mills in which the material is subjected to complex loads, the main component of which is shear loads [27,28,31], no adequate models have been developed so far to describe the relationship between the comminution energy, process parameters and size reduction ratio. As indicated in previous papers [1,27,28,29,32], multi-disc mills have high efficiency and are well suited for the grinding of biological fibrous materials because of the occurrence of shear loads [1].

In this paper, the research was conducted for rice and corn comminution. Rice and corn play a significant role in the agricultural and food sector [33,34]. These are currently the most widely used grains by many worldwide industries [35,36,37]. It is estimated that rice and its products are the staple food for more than 1/3 of the global population [33], while corn occupies the largest area among all arable crops [38]. In the case of both these grains, comminution is used for obtaining flour, flakes, and semi-finished products for human nutrition and also for the preparation of animal feed [39]. Hence, it is reasonable to develop comminution models for these materials, which will contribute to improving the design of grinding mills as well as increasing the quality of the ground product and reducing the final energy consumption [40].

Considering the above, the purpose of this research is to develop models for the particle size distribution of the ground product in multi-disc mills depending on the variable process parameters, i.e., disc rotational velocity and, consequently, power consumption, and the relationship between the grinding energy and the shape of particle size distribution curves, which would help predict the product size reduction ratio. In addition, the developed models would provide a basis for the numerical modelling of the grinding process using disc mills.

## 2. Materials and Methods

In this study, the relationships between the distribution coefficients and the average angular velocity of grinder discs, power consumption, and specific comminution energy were determined for rice and corn grinding using the RWT-KZ5 five-disc mill. The comminution process in this study was oriented on the size reduction of selected materials for various purposes. Detailed experimental procedures and methodology are described in the sections below.

### 2.1. Materials

Rice and corn were used as the experimental materials in this study. Grains selected for tests were procured from local stores in tightly sealed packages. Before comminution, the moisture content of grains was established using the gravimetric method [41]. In this method, grains are dried at 105 °C, and the wet basis moisture content is determined as follows [42]:

*W_m_* = ((*m*_1_ − *m*_2_)/*m*_1_)·100%, (1)

where *W_m_* is the total moisture of the sample, %, *m*_1_ is the mass of the sample before drying, g, and *m*_2_ is the mass of the sample after drying, g.

Moisture content was measured using the MAC 210/NP moisture content analyser (RADWAG, Radom, Poland), capable of determining the moisture content with an indication error of 0.001%. The moisture content of grains was 12.23% for corn and 11.34% for rice.

### 2.2. Test Stand and Experiment Set Up

**Figure 1:**
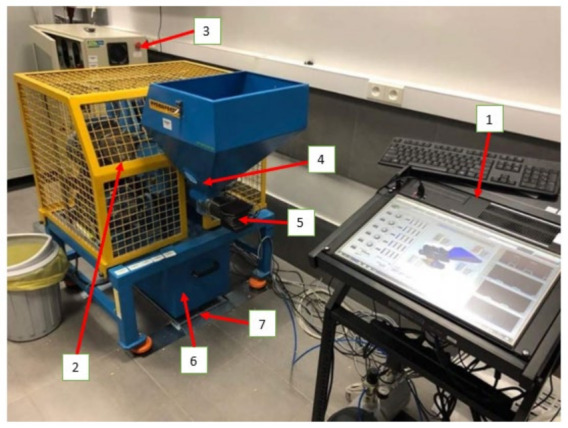
Test stand of a multi-disc grinder: 1—control unit, 2—multi-disc grinder, 3—control cabinet, 4—screw feeder, 5—step motor, 6—receiving basket, 7—scale [43].

In this paper, the relationship between the process parameters and the size reduction ratio was examined in the example of a multi-disc mill, more specifically, the RWT-KZ5 five-disc mill (Figure 1).

The RWT_KZ_5 five-disc mill (Figure 1) test stand available at the University of Science and Technology in Bydgoszcz, Bydgoszcz, Poland, is equipped with the ADP 8080 control system (ADP, Brzoza, Poland) with a PC, a batch material feeder with a screw conveyor, for which the feeding speed is controlled by a step motor, a receiving basket and product scales (ADP, Brzoza, Poland), and a product particle size and shape identification system. The stand is controlled by the control panel using the Młyn 2019 application operating in the LABView 2012 environment (National Instruments, Austin, TX, USA). The design of the stand and control system are discussed in detail in previous publications [43,44].

**Figure 2:**
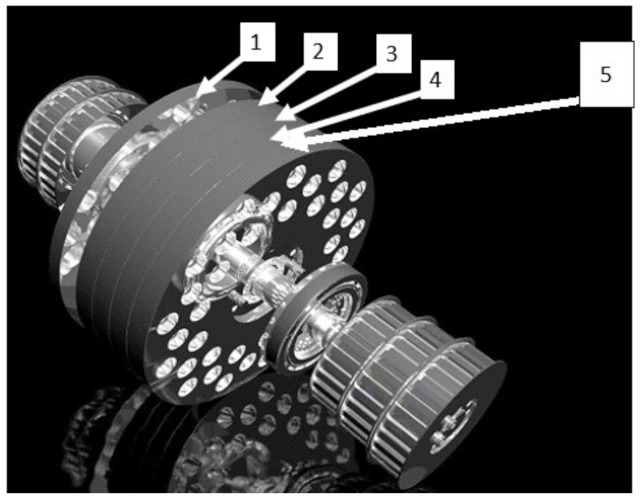
A schematic of the comminution unit structural features; numbers from 1 to 5 represent successive discs [43].

A characteristic and innovative feature of the five-disc mill is the design of its working unit and drive unit [45]. The mill working unit consists of five discs with holes of different diameters in each of the discs and different hole spacing (Figure 2), as shown in Table 1. The discs are installed on the same shaft and placed in the comminution chamber at an angle. Each disc has an individual electric drive controlled by the control panel using an inverter so that the rotational velocity can be adjusted independently for each disc.

Thanks to the applied control and monitoring system [43,44], the following parameters are recorded and archived in real-time during grinding: angular velocity, torque and power for each disc, batch moisture content, mass of comminuted product, and product particle size.

For the purposes of determining the relationship between energy consumption and process parameters, the angular velocity of individual discs was considered a process variable. The presented program of tested angular velocity settings (in Table 2) was based on ref. [21,43] as a continuation of the tests of functional characteristics of the multi-disc biomass grinder for implementation purposes. The differences in the five test programs (I to V) are the result of choosing different gradients of disc speeds in each program. In the first program, the angular speed increased from the first disc with angular speed set as 20 rad/s to the last disc and the difference in angular velocities between each disc was equal to Δ*ω*. In the second test program, the angular speed of the disc increased from the fifth disc with the angular speed set as 20 rad/s to the first disc and the difference in angular velocities between each disc was equal to Δ*ω*. Each configuration within the first and second test program indicated a different value of Δω. In the third test program, the angular velocities were set alternately: smaller by Δω and larger by Δω, and in each configuration, the initial value of the angular velocity of disc 1 was higher than in the previous one. The fourth program assumed that the speeds on the disks would be alternating: higher, lower by Δω, with each configuration different in the speed difference Δω (larger for subsequent configuration) between the disks. In the fifth research program, the angular velocities of the disks were set alternately: smaller–greater by Δω, while in subsequent configurations, the difference Δω between the angular velocities of the disks was reduced.

One kg of rice and corn grains was comminuted at each disc setting. The time, change in angular velocity, power consumption, torque for each disc, and the mass of the comminuted material were recorded at a sampling interval of 0.5 s. After comminution, the particle size distribution of the ground material was determined. For this purpose, particle size analysis with the use of digital image processing on the Camsizer (Retsch Technology GmbH, Haan, Germany) was conducted. This involved samples of 50 g.

The relationship between specific comminution energy and the product particle size was also determined. Specific comminution energy in comminution machine tests is described as follows [1]:

(2) $$E_{s}=\frac{1}{m}\int_{t_{0}}^{t}\left(P_{c}-P_{i}\right)dt,$$

where *m* is the grinding product weight, kg, *P_c_* is the total power consumption during grinding, kW, and *P_i_* is the idle power consumption, kW.

For a five-disc mill, the above equation can be written as:

(3) $$E_{s}=\frac{1}{m}\int_{t_{0}}^{t}\left(\left(P_{c1}+P_{c2}+P_{c3}+P_{c4}+P_{c5}\right)-\left(P_{i1}+P_{i2}+P_{i3}+P_{i4}+P_{i5}\right)\right)dt,$$

where *P_c1, 2, 3, 4, 5_* is the total power consumption during grinding for disc 1, 2, 3, 4, and 5, respectively, kW, and *P_i1, 2, 3, 4, 5_* is the idle power consumption for disc 1, 2, 3, 4, and 5, respectively, kW.

The material size reduction after comminution was described by the size reduction ratio of the feed grain size to the product grain size material, and 80% of the tested sample mass (volume) was below it, as determined according to the results of granulometric analysis [43,46]:

(4) $$\lambda_{80}=\frac{D_{80}}{d_{80}},$$

where *D*_80_ is the dimension of the sieve hole, through which 80% of the feed material passes, and *d*_80_ is the dimension of the sieve hole, through which 80% of the grinding product passes. This paper describes the relationship between the specific comminution energy and size reduction ratio.

### 2.3. Particle Size Ditribution

**Figure 3:**
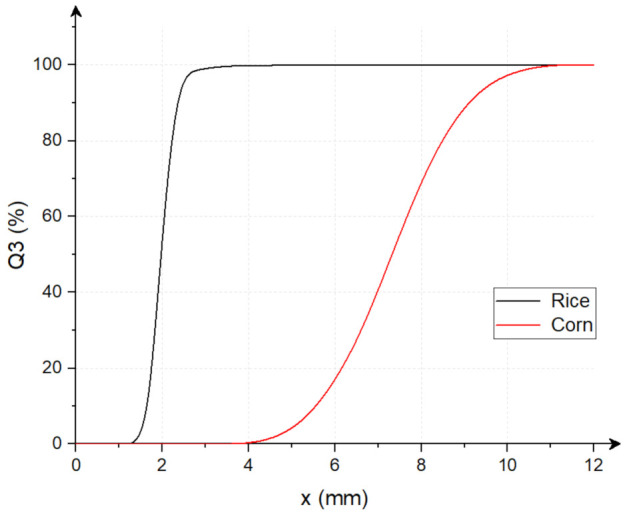
The cumulative particle size distribution of rice and corn grains before comminution; Q3—cumulative size distribution (based on volume), x—particle diameter, which is the shortest chord of the measured set of maximum chords of a particle projection.

Particle size analysis was conducted for both the feed material and the ground product. The Camsizer (Retsch Technology GmbH, Haan, Germany) was used for this purpose, following the ISO 13322-2: 2006 standard [47]. Figure 3 show the cumulative size distributions of rice and corn grains before comminution.

For the ground samples, the cumulative particle size distribution was approximated using a two-parameter Weibull distribution. The Weibull distribution, more precisely the Rosin–Rammler–Sperling–Bennet (RRSB) distribution, is very often used to describe particle size distributions, in particular those in which very small particles are dominant [11,48], and therefore it was used for this paper. The RRSB distribution describes the relationship as [11,48,49]:

(5) $$Q(x)=1-\exp{\left(-\frac{x}{x_{o}}\right)}^{k},\;\;\;\;\;\;x\in \left(0,\infty \right),$$

where *x* is the relative or absolute particle diameter, *x*_0_, and *k* are the distribution parameters based on experimental data. The parameter *x*_0_ reflects the particle size for which the cumulative particle size distribution is 0.632 [48] and is also known as the statistical average diameter [49]. The *k* parameter determines the heterogeneity of the particle distribution in relation to the statistical average diameter; the lower the *k*, the greater the homogeneity of the particle size distribution around the statistical average diameter [48,49].

RRSB distributions were approximated using OriginPro 2022b software (OriginLab Corporation, Northampton, MA, USA) and the Lavenberg–Marquardt algorithm.

## 3. Results and Discussion

### 3.1. Power Consumption and Specific Energy Demand

Table 3 summarize the results of power consumption and specific energy demand at the tested settings of disc angular velocity. The specific comminution energy was calculated based on the power consumption and ground product weight measurement according to Formula (3). The summary in Table 3 show the final values of the comminution power consumption *P_c_* and the idle power consumption *P_i_*. Detailed data on power consumption for each of the discs are presented in Tables S1–S3.

The results presented in Table 3 indicate that the idle power consumption, the total power consumption, and the specific comminution energy depend on the disc angular velocity. It can be noticed that a higher disc angular velocity increases the power consumption, both when idle and when grinding the material. It is similar in the case of specific comminution energy: an increase in disc angular velocity causes an increase in specific comminution energy, which demonstrates that the dynamics of increase in power consumption when changing the angular velocities is much greater than the dynamics of changes in the comminution efficiency. Details on the dependency of power consumption, efficiency, and specific comminution energy on process parameters such as the angular velocity of discs, batch feed speed, and the number of discs involved in grinding for a five-disc mill were presented in previous papers [16,19,24], and therefore they will not be discussed here in detail.

It should be noted that idle power consumption is a major part of the total power consumption (54 to 85% depending on configuration, see Table 3). Hence, it is necessary to improve the grinder design to reduce the idle run and increase the mechanical energy efficiency. It was noted that corn grains required higher energy inputs for grinding compared to rice in all tested configurations of the angular velocity of discs.

**Figure 4:**
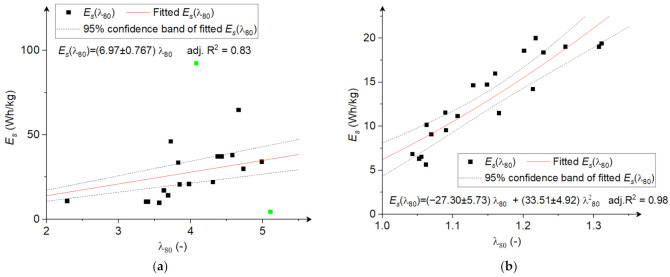
The dependence between size reduction ratio and specific comminution energy: (**a**) corn grains; (**b**) rice grains. The green points are outliers and not included in the analysis.

Figure 4 show the relationship between the size reduction ratio and specific comminution energy during the grinding of rice and corn grains. In both cases, the specific comminution energy increased with the size reduction ratio. Similar relationships were observed for other types of grinders used for granular materials, such as ball mills [14], hammer mills [1,17], knife mills [50], and roller mills [15,16]. An increase in comminution energy with an increase in size reduction ratio is due to the fact that with the decreasing size of ground material, the comminution energy increases as the internal flaws decrease (with the size decrease); thus, breakage probability is reduced, and subsequent size reduction involves higher energy input [1]. This agrees well with known comminution laws (Bond’s, Kick’s, Rittinger’s, and others being their modification), generally agreeing with the variation of energy-based law proposed by Walker et al. [51], which predicts energy consumption increase with the decrease in particle size [52].

For the purpose of developing fitting curves describing the relationship between size reduction ratio and comminution energy, it was assumed that E_s_ ∈ (0, ∞), and therefore the intercept was set as constant and equal to 0. In this case, the relationship between the size reduction ratio and specific comminution energy during the grinding of corn grains had a linear relationship (adj. R^2^ = 0.83) (Figure 4a) or could also be described by a quadratic function (Figure 4b) when grinding rice with the determination coefficient adj. R^2^ = 0.98. To a large extent, the form of the function describing the relationship between the size reduction ratio and specific comminution energy depends on the properties of the material to be comminuted, such as moisture content, initial size, and strength properties, as demonstrated in this study, where completely different forms of fitting functions were obtained for two materials ground in the same mill. Similar conclusions are presented in ref. [1], where different fitting functions were obtained for materials with different moisture content to describe the relationship between the particle size after comminution and the specific comminution energy.

As presented in Figure 4, the size reduction ratio of rice was low, and the dimensions were reduced only slightly during grinding. In the case of corn, the size reduction ratio was even more than double that for rice at similar comminution energy levels. Given the above, it should be concluded that the multi-disc design in the current configuration and at the tested angular velocity range (20–100 rad/s) is better suited for grinding corn grains for size reduction processes. Adapting the mill design to rice grinding may involve reducing the gap between the discs, which could increase the size reduction ratio and result in obtaining finer particles. As shown in ref. [53], a decrease in disc gap results in an increase in size reduction ratio; however, it also increases energy consumption. The study presented by Yildirim et al. [54] also confirms this assumption, while it was shown that increasing the gap between grinding discs led to a higher amount of coarse particles in the ground product. Other possible changes to improve the size reduction of rice could be reducing the hole diameters and increasing the number of holes on the disc circumference to intensify and increase the number of contacts between the mill’s cutting edges and the material. In addition, increasing the disc rotational velocities could also improve rice grinding by preventing unground material from flowing through the holes and increasing the number of contacts between particles and cutting edges. This specific recommendation could be justified by the nature of the grinding mechanism in the multi-hole, multi-disc mill, which is slightly different from a typical disc mill. In a typical disc mill, the friction forces are mainly responsible for grain disintegration and attrition [55]. In the proposed design of a multi-hole multi-disc mill, grains are subjected to a complex state of stress where a shearing (cutting) mechanism is dominant between the edges of holes in subsequent discs and as well as impact compression [56]. The holes in the discs have two functions: allowing the flow of material between the discs and grinding the material. The grains fed to the grinding chamber fill the holes in the first disc. Grinding occurs in the common area created when the holes from the second and then subsequent disc move relative to the holes in the first disc (and subsequent) and materials placed between two shearing edges of holes break. The common area created by subsequent holes changes over time. The broken particles with a size equal to or smaller than the disc gap leave the holes and the grinding unit through the gap between discs, where they can also be secondarily fragmented by friction force occurring during contact with rotating disc walls. The volume of the material which will pass through the disc is dependent on the hole diameter, hole number, and hole distribution around the perimeter of the disc and angular velocity of the disc, which will determine the time of filling the holes [57]. The time of filling the holes is dependent as well on the particle velocity, which is a result of gravity and horizontal and vertical velocities forced by the contact of a particle with walls of holes in a rotating disc, the dosing stream of material from the feeder and particle size (the smaller the particle, a higher amount can be placed in the hole) [58]. As was shown in ref. [59], in this type of grinding unit design, the material column being cut is not the entire material that has been moved to the lower hole. Its volume is a product of holes momentary common cross section created by the holes of subsequent discs (dependent on time) effectively used for cutting and the height of material column filling the hole. The entire material moved from the preceding hole to the subsequent hole is higher than this subjected to shearing and involves a mixture of ground and unground material. Decreasing the diameter of holes to adjust the design of the mill for obtaining the finer particles, will then result in decreasing the layer of material subjected to cutting and decreasing the grains flowing through the hole without contact with hole comminution edge. This will increase the probability of impacts and grains shearing during passing the subsequent discs. Increasing the number of holes will also increase the number of impacts, similarly to increasing the angular velocities leading to generation of finer particles during grinding. As it was shown for other mills [60,61], increasing the speed of rotation (in consequence the number of impacts) results in decreasing of particle sizes. It is worthwhile to note that decreasing the holes diameter and increasing the angular velocities could only be carried out to certain critical values, determined experimentally, which implies the necessity of further research on this topic.

### 3.2. Rosin–Rammler–Sperling–Bennet Distributions for a Multi-Disc Ground Product

**Figure 5:**
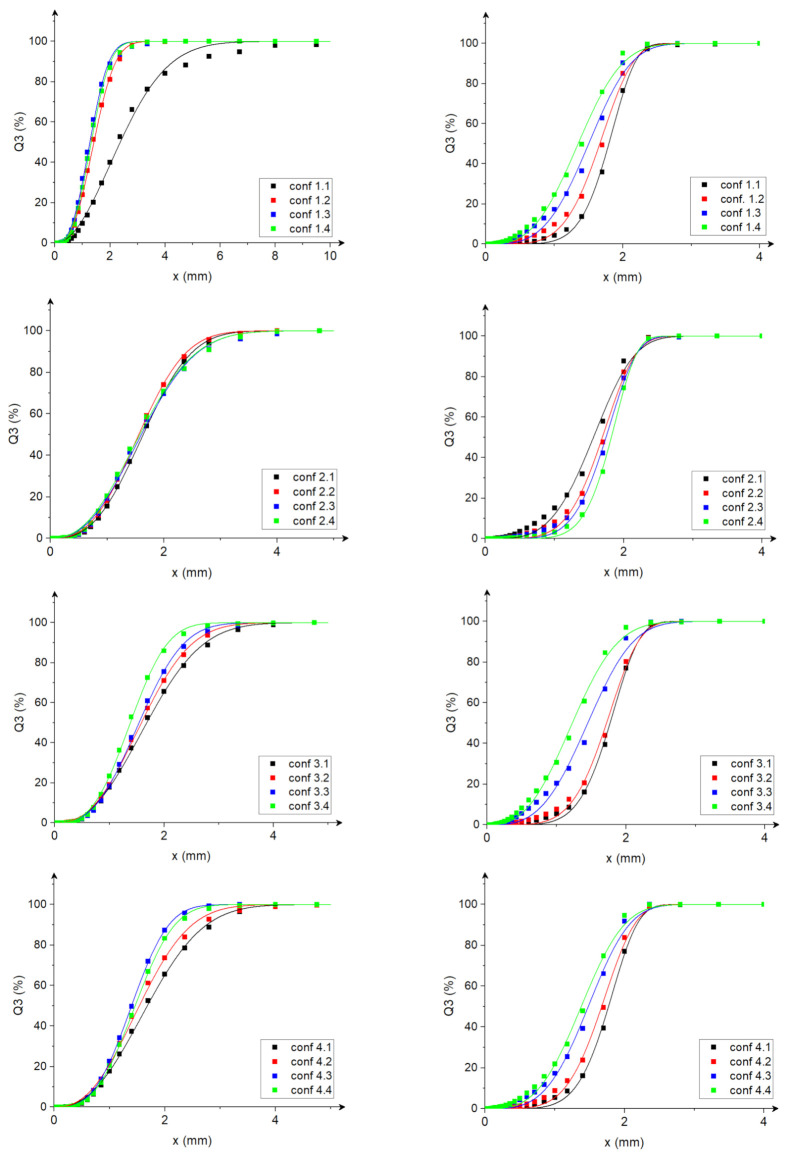

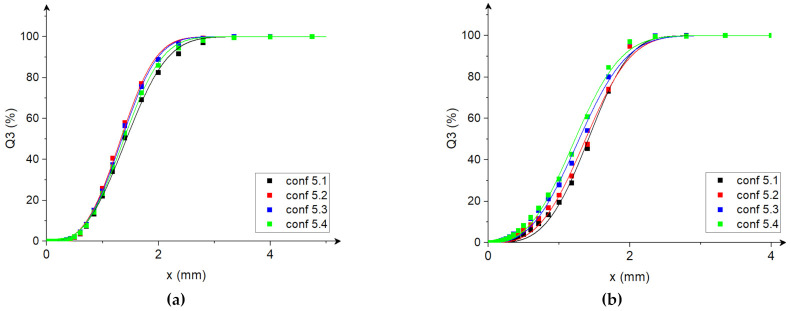
Cumulative particle size distributions of the ground product for the tested configurations of disc angular velocity settings with the fitted RRSB distribution curves; (**a**) corn grains and (**b**) rice grains. The solid lines are the fitted RRSB distributions, conf. means the configuration of the disc setting.

An analysis of particle size distribution was conducted for each of the ground materials tested at different disc angular velocity settings in the multi-disc grinder (as per Table 2). Figure 5 show the experimentally determined cumulative particle size distributions of the grinding product with fitted RRSB distribution curves. When looking at the cumulative particle size distribution curves, we can observe a higher content of fine fraction (particularly below 0.5 mm) in the comminuted corn samples compared with rice samples. This confirms the earlier observation of a lower size reduction ratio for rice. The shape of the particle size distribution curve also noticeably changed, and so did the angular velocity of the mill discs (equivalent to the change in the disc angular velocity settings in Figure 5). Generally, increasing the angular velocities of the five-disc mill resulted in a higher probability of producing smaller-size particles, which is associated with the intensification of comminution by increasing the number of contacts between the grinding edge and the grain. Published studies have indicated that with an increase in peripheral speed, the particles would pass through the comminution zone at a higher frequency during a given time period [62]. Similar results were reported by Kratky and Jirout [62] and Bitra et al. [63].

The curves presented in Figure 5 are RRSB distribution curves fitted to the experimental data. Table 4 present coefficients *x*_0,_ *k*, *μ* (average value), *σ* (variance), and adj. R^2^ for these distributions. For all distributions, the determination coefficient adj. R^2^ was higher than 0.99, which proves a very good fit of the theoretical distributions to the experimental data. Therefore, it might be concluded that the Rosin–Rammler–Sperling–Bennet distribution can be used to describe the particle size distribution of biomaterials ground in a multi-disc mill. Coefficient *x*_0_ for RRSB distributions for the tested configurations was in the range of (1.37–2.84) and (1.40–1.90) for rice samples and corn samples, respectively. The average value of distributions was in the range of (1.29–2.52) and (1.25–1.94) for corn and rice, respectively. For most configurations, both the average value of distribution and coefficient *x*_0_ (interpreted as the particle size for which the value of the cumulative distribution is 63.2%) were lower for corn, which proves better comminution, e.g., higher content of fine particles than in the case of rice. It is important to note that the ranges of *x*_0_ and *μ* coefficients for rice and corn coincide, which indicates certain characteristics of grain grinding; it might be concluded that these coefficients define a certain characteristic range of resulting particle sizes (regardless of the initial size of the grains) for the analysed multi-disc grinder design in the tested range of disc angular velocities and the current configuration of the arrangement and size of holes in the discs.

Coefficient *k* determining the shape of the distribution curve was in the range of 2.00–3.06 for corn and 2.13–6.87 for rice. For the ground corn samples, the shape of the distribution is similar to the Rayleigh distribution considering the k values close to 2. This indicates that the particle size in the density function is concentrated on the left side of the distribution, i.e., closer to 0, which indicates higher content of fines fraction when grinding corn in a multi-disc mill. For rice samples, the values of coefficient *k* vary much more depending on the configuration, as proven by the wide range of values of this coefficient (2.13–6.87). Higher values of *k* show a right shift of the density distribution, which means moving away from the initial value. In addition, for rice, we can observe a greater impact of the angular velocity settings of discs on the shape of the RRSB distribution curve than in the case of corn comminution. In the case of rice comminution, for configurations with low angular velocities, the values of coefficient *k* were greater (see Table 4) than for the settings with higher angular velocities. Interestingly, it was the opposite for corn in the case of higher angular velocities, where lower values of coefficient *k* were recorded.

The values of distribution variance *σ* were in the range of 0.48–1.31 for corn and 0.31–0.50 for rice. Distribution variances for the ground rice samples had lower values than the variances for the ground corn samples. This indicates a more frequent occurrence of particle sizes close to the average value and a lower spread of the particle size range for the rice samples. It was observed that, in the case of rice samples, the distribution variance for the settings with high values of disc angular velocities was lower than for the settings with low values of disc angular velocities. In the case of corn samples, the distribution variance for the settings with high values of disc angular velocities was higher than for the settings with low values of disc angular velocities.

### 3.3. Relationships between the Disc Angular Velocity, Power Consumption, and Specific Energy Demand and the Shape of the Particle Size Distribution of the Grinding Product

Correlation analysis using the Spearman method was conducted to determine the relationship between angular velocities, power consumption, and specific comminution energy with the RRSB distribution parameters (Table 5). A two-sided significance test was carried out with a confidence level of 0.05. It was demonstrated that parameters *x*_0_, *k*, *μ,* and *σ* are correlated with the average angular velocity of the miller discs, power consumption, and specific comminution energy. For corn comminution, negative correlations occurred between *x*_0_, *μ,* and *σ* with average angular velocity, power consumption, and specific comminution energy. However, parameter *k* presented a positive correlation with these parameters. For rice comminution, parameters *x*_0_, *μ,* and *k* were correlated negatively, while *σ* was correlated positively with the average angular velocity, power consumption, and specific comminution energy. All the observed correlations were significant, and in all cases, the coefficient determining the correlation strength was greater than 0.6. The correlation coefficients between the variables were generally lower for the corn comminution process than for rice due to nonuniformity in the grinding process. This phenomenon was described in detail in previous works [21,64,65], showing a variable efficiency and corn grains having problems with passing through and moving outside the comminution chamber, which had an impact on obtaining different efficiency and specific comminution energy values.

**Figure 6:**
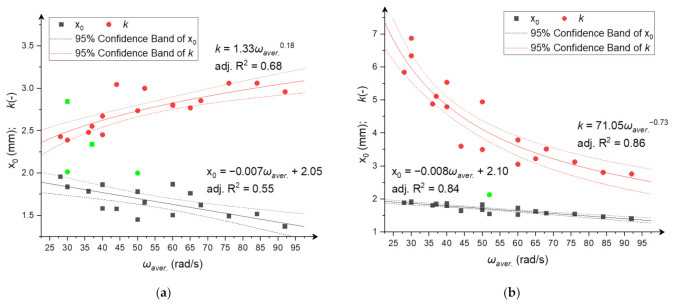
The dependence between RRSB distribution parameters and average angular disc velocities during comminution; (**a**) corn grains; (**b**) rice grains. The green points are outliers not included in the analysis.

**Figure 7:**
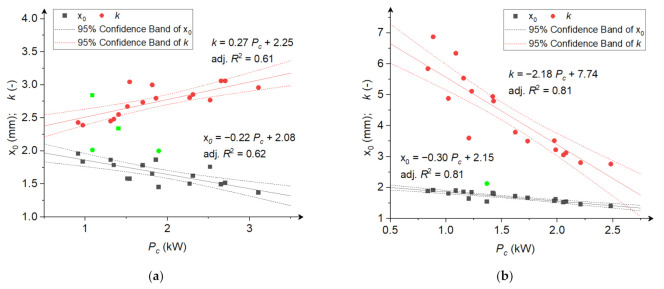
The dependence between RRSB distribution parameters and power consumption during comminution; (**a**) corn grains; (**b**) rice grains. The green points are outliers not included in the analysis.

**Figure 8:**
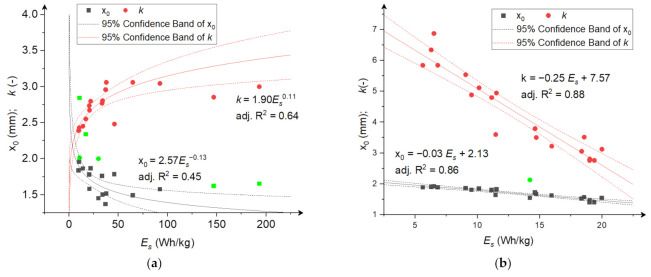
The dependence between RRSB distribution parameters and specific comminution energy; (**a**) corn grains; (**b**) rice grains. The green points are outliers not included in the analysis.

**Figure A1:**
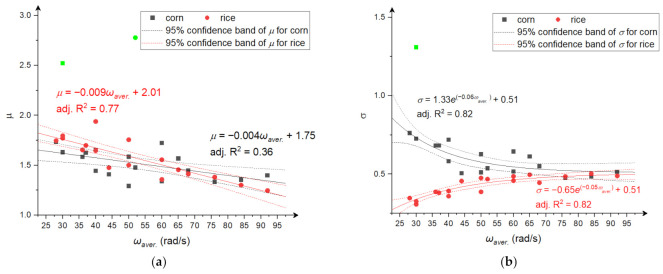
The dependence between *μ* (average value) and *σ* (variance) and average angular disc velocities during comminution; (**a**) *μ* (average value); (**b**) *σ* (variance). The green points are outliers not included in the analysis.

**Figure A2:**
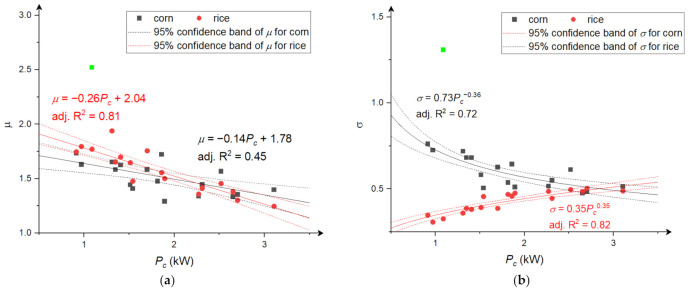
The dependence between *μ* (average value) and *σ* (variance) and power consumption during comminution; (**a**) *μ* (average value); (**b**) *σ* (variance). The green points are outliers not included in the analysis.

**Figure A3:**
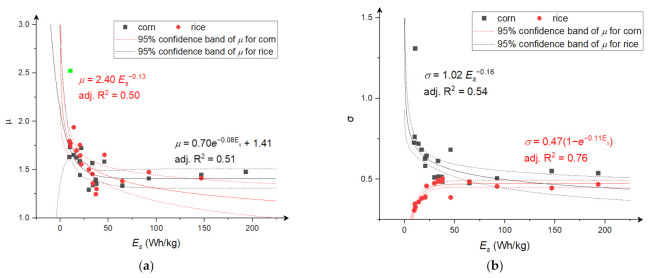
The dependence between *μ* (average value) and *σ* (variance) and specific comminution energy; (**a**) *μ* (average value); (**b**) *σ* (variance). The green points are outliers not included in the analysis.

Figure 6, Figure 7 and Figure 8 show coefficients *x*_0_ and *k* of the RRSB distributions as a function of the average angular velocity (Figure 6), power consumption (Figure 7), and specific comminution energy (Figure 8). Figure A1, Figure A2 and Figure A3 in Appendix A also show the relationships between the expected value of *μ* and variance *σ*. The dependence of coefficient *x*_0_ on the average angular velocity and power consumption for both grinding products and the specific comminution energy for rice comminution can be described by a decreasing linear function (Figure 6, Figure 7 and Figure 8b). The relationship between coefficient *x*_0_ and the specific comminution energy for the corn comminution process can be described with good fitting (adj. R^2^ = 0.64) using a power function (Figure 8a).

The relationship between shape coefficient *k* for the corn comminution process and the average disc velocity and specific comminution energy can be described with good fitting (adj. R^2^ > 0.6) using an increasing power function (Figure 6a and Figure 8a). The relationship between coefficient k and power consumption can be described by a decreasing linear function for corn comminution and a decreasing linear function for rice (Figure 7). For the rice comminution process, the relationship between the disc angular velocity and coefficient *k* is well described by a decreasing power function (adj. R^2^ = 0.86, Figure 6b). The linear model with a negative linear coefficient (Figure 8b) explained 88% of the changes in shape coefficient k depending on the specific comminution energy. It should be noticed that the *k* parameter values of fitted distributions were higher for ground rice particles than for ground corn. This shows that the homogeneity of particle sizes was larger (with a narrow distribution) for ground corn. This is the result of the different nature of stresses which dominate during rice and corn comminution. As previously mentioned, two types of stresses occur in the tested mill type: shearing and impact compression. As presented in ref. [1], subjecting the material to impact stress in the mill will lead to a wider particle distribution of ground material, while shearing (cutting) stresses in the mill will produce a product with narrow particle size distribution. This leads to the assumption that shearing loads are dominant during corn grinding in the analysed multi-disc mill, while impact loads are dominant during rice comminution. This is the effect of the ratio of corn and rice kernel size and the gap between the grinding discs. The ratio between the gap dimension and rice grain dimension is low, so the grains are mostly ground between the first two discs to a size lower than the gap dimensions, and further size reduction is a result of impact. Size reduction due to impact in the case of rice can take place as well during flowing through holes, because the forces and energies for rice damage are relatively low [66], lower than corn [40]. The dominant stress in the mill as the resultant of the materials’ internal structure and mechanical properties, also affect the size reduction. Corn with a fibrous seed coat and layer of horny endosperm shows lower resistance to shearing than impact, and more glassy rice (in the same showing more brittle characteristics) will be crushed by impact rather than shearing. The increase in value of size distribution parameter k with the increase in angular velocity (and, in consequence, increase in power and specific energy consumption) in the case of ground corn may result from an increase in the share (influence) of impact stresses during corn grinding along with an increase in angular velocities. The reason for the decrease in parameter *k* of the particle size distribution of the ground rice with increasing angular velocities, power consumption, and specific energy consumption can be indicated, in turn, as the increase in the number of contacts with increasing angular velocities, leading to multiple particle refinement and obtaining a more homogeneous product. The dependencies shown in Figure 6, Figure 7 and Figure 8 and Figure A1, Figure A2 and Figure A3 clearly indicate a strong relationship between the parameters of product particle size distributions and angular velocities, power consumption, and grinding energy. The developed models can be a source of information for numerical modelling of comminution processes.

It is worthwhile to note that obtaining a product with smaller particles involves higher energy expenditure and a greater comminution speed and number of impacts, which is provided by increasing the angular velocity of the discs in the disc mill used in this study. Higher energy consumption for obtaining the finer product was also observed for hammermilling [67]. This is consistent with Brach’s comminution theory, which indicates that comminution energy in subsequent comminution cycles is a multiplication of energy for the first breakage [68]. This also has an impact on the shape of the distribution curve and the variance describing the distribution of particle size around the expected value (average particle size in the sample).

Previously conducted research on different materials comminution for both brittle and biomaterials using different types of mills showed that the relationship between energy and ground material particle size is dependent on the comminution machine features, operation parameters (such as rotating speed of comminution elements), and material characteristics [1,69]. In the studies of ref. [69,70], the energy–size reduction model for ores comminution was proposed, which indicates that the breakage energy is affected by the material properties (hardness), machine type, and particle size exponent that is a function of size. It should be noted that the material characteristics that need to be taken into account in the case of biomaterials are much more than hardness.

There exist some differences between the breakage mechanism of biomaterials such as cereal grains the synthetic or other types of brittle materials. The characteristic of brittle materials during fracture are low deformation, low impact resistance, high compression, and low tensile strength [71]. This behaviour is typical of most inorganic non-metallic materials [71]. The grains such as corn, rice, wheat, and soybean are materials that shows more elastic and plastic behaviour than the brittle inorganic materials [72,73]. It was shown that most agricultural materials reveal elastic behaviour in the first loading part, and with the increased loading, the properties change into viscoelastic [74]. In addition, the behaviour and impact resistance of the grains changes with several properties such as grain size, strength, vitreousness, the internal structure (including the share of starch, and protein), hardness, and moisture content [75,76,77]. A higher moisture content makes the grains softer and more plastic which leads to larger deformations in comparison with grains at a low moisture content [72,73]. Additionally, the internal structure and composition of the grain will impact the breakage behaviour and breakage energy, as well as the particle size after breakage. Moreover, varietal differences also influence the breakage behaviour of grains due to their differences in composition [78,79,80,81,82,83,84]. For example, in corn grains, the horny endosperm show lower deformation and can withstand higher forces when compared with floury endosperm and germs [85].

Comparing the relationships for two different types of grains, we can clearly see the difference between the approximating functions and their coefficients. This is partially caused by the differences in the internal structure of rice and corn grains and the resulting susceptibility to impact and shear loads, as well as the ratio between the grain size and the working gap and the size of holes, which determines the grinding and the flow of material between the mill discs. In previous studies, it was shown that hard biological materials need higher energy input in comminution than soft ones [34,35,36]. An increase in vitrousness and moisture content leads to an increase in force and energy demand in the process of grain comminution [79,80,81,82,83,84]. Generally, in the case of hard materials, finer product can be obtained [86]. Looking at the difference in the corn and rice grain structure and the grinding mechanism, it should be pointed out that white rice was used (after bran and germ removal), so mainly the endosperm was subjected to grinding, which has high glassy properties. Rice is characterised by a more vitreous structure than corn, which makes it very brittle. The corn kernel was comminuted with the bran and the germ. As was shown in the example of wheat [87], in the first stage of grinding the kernel is broken for separating the bran from endosperm. This takes place for corn when grains pass through the first pair of discs. Corn has a harder exterior seed coat, while its interior structure is more mealy and soft (floury), which means comminution can take place when material merely hits the disc (with lower impact forces, velocities, energies), even without cutting. The coarse particles are then produced from the bran and the horny endosperm, while the finer from floury endosperm [85]. The increasing velocities and, in consequence, the energy of impacts results in the higher fragmentation degree of harder parts of corn grains [85].

The relationship between the initial grain size and the grinding holes is also different for rice and corn grains. Rice grains are smaller than corn grains, so the grain size to hole size ratio is smaller for rice, which at low disc angular velocities may cause some of the rice grains to pass freely through the space created by the holes in adjacent discs without being ground. This is a probable reason for the low size reduction ratio values obtained for rice, especially for settings with low angular velocities. The other reason is the ratio between grain size and the gap between the discs. The gap between the disc allows the particles to leave the comminution zone, so the grains with particles smaller than the gap will be discarded and not ground further. As was shown in ref. [62], the particles with sizes smaller than 1 mm are more likely to be thrown outside the comminution zone due to particle acceleration caused by the rotating elements, so a small amount of particles below 1 mm is observed in the comminution product for both rice and corn. As the rice grains are smaller than corn grains, they are reduced in size to dimensions lower than the gap after one or two passages between subsequent discs and leave the comminution zone, so the power consumption and the specific energy consumption are lower due to the low portion of the material being ground in the same time period. These irregularities in batch between discs are visible in the changes of power consumption and torques in time and were described for this mill in one of the previous studies [65]. So, for rice, mainly the increase of size reduction (and getting smaller particles) with increasing angular velocity is the effect of the higher energy of the first impact and higher impact probability during passing between the first two discs. This is visible, especially when analysing the power consumption individually on each disc (Tables S1–S3, Supplementary Materials), while in the case of rice, the power consumption increases the most on the first and second disc while the power consumption increases only slightly from the level of idle run.

The results reported the influence of moisture content on the formation of fine and coarse particles as well as size reduction during grinding. The increase in moisture content caused the increase in breakage rate during hammermilling of corn [88]. For the five-disc mill, it was shown in the example of rice that increasing the moisture content leads to an increase in the size reduction ratio [89]. In general, the increase in MC led to an increase in the formation of coarse particles during comminution and a decrease of fine particles due to the decrease of hardness and brittleness with moisture content [90,91].

The results presented also demonstrate the need for grain specific approach to understanding the comminution process of cereal grains. It has also shown the variety of shapes of particle size distribution curves for different grains, even when the same process parameters or energy levels are used for grinding material. The study confirms earlier conclusions from other research [1,69] that the particle size distributions are affected by the machine design, machine operation parameters, materials properties, and energy–particle size distributions. The model presented in this research predicts the behaviour during comminution during grains for a given design of the mill. Creating a universal model for mills designed for grinding biomaterials with very different characteristics, considering the design diversity of the mills, is a challenge, requiring many experiments for various structures and materials with different parameters, e.g., humidity, hardness, and should be the focus of further research on the development of energy–size relationship for biomaterials comminution.

## 4. Conclusions

This paper presents the relationships between the particle size distribution of ground materials with the angular velocity of the cutting pieces in a mill with multiple multi-hole disks. In addition, the relationships between the machine power consumption and specific comminution energy, particle size and particle size distribution curves were also presented.

From the results, it was determined that the idle power consumption, total power consumption, and specific comminution energy depend on the disc angular velocity settings in such a way that increases the disc angular velocities and causes an increase in these parameters. It has also been demonstrated that the specific comminution energy increases with the size reduction ratio.

Regardless of the considered setting of disc angular velocities for the RRSB distributions, the adj. R^2^ was higher than 0.99, which proves a very good fit for the experimental data. Therefore, it might be concluded that the RRSB distribution can be used to describe the particle size distribution of biomaterials subjected to comminution in a multi-disc mill.

It was demonstrated that a negative correlation occurred between *x*_0_, *μ,* and *σ* and average angular velocity, power consumption, and specific comminution energy, and a positive correlation between parameter *k* in the case of corn grinding. In the case of rice, a negative correlation occurred between *x*_0_, *μ*, and k and average angular velocity, power consumption, and specific comminution energy. These relationships were presented in the form of linear and power functions.

Given the presented relationships concerning the fine particle content and size reduction ratio, it should be concluded that the multi-disc design in the current configuration and at the tested angular velocity range (20–100 rad/s) is better suited for grinding corn grains. To improve the size reduction ratio of rice during multi-hole multi-disc milling, it is recommended to decrease the gap between discs, increase the number of holes, and decrease its diameter; however, further research is required to establish the relationship between these design features of discs, and the shape of the grain distribution curves and specific comminution energy.

In designing the comminution equipment and predicting the particle size distribution, the materials properties, mill design features, and consequently the process parameters should be taken into account because they all have an influence on the energy demand and share of fine particles in the ground product.

## Figures and Tables

**Table 1:** Design details of comminution discs.

Parameter		Unit	Disc 1	Disc 2	Disc 3	Disc 4	Disc 5
Disc diameter		mm	274	274	274	274	274
Number of holes			14	22	27	33	39
Hole diameter		mm	30	23	21	17.5	17.5
Number of rows with holes			2	2	3	3	3
Number of holes in a row			7	11	9	11	13
The radius of the arrangement of the holes in the row	R1	mm	101.7	107.4	110.8	114.8	117
	R2	mm	85	82.3	95.8	99.8	102
	R3	mm	-	-	79.8	79.8	82

**Table 2:** Tested configurations of batch dosing speed and comminution disc velocities [21,43].

Test Program	No. of Configuration	*ω_aver._*	∆*ω*	*ω*_1_	*ω*_2_	*ω*_3_	*ω*_4_	*ω*_5_
		Rad·s^−1^	Rad·s^−1^	Rad·s^−1^	Rad·s^−1^	Rad·s^−1^	Rad·s^−1^	Rad·s^−1^
I	1	30	5	20	25	30	35	40
	2	40	10	20	30	40	50	60
	3	50	15	20	35	50	65	80
	4	60	20	20	40	60	80	100
II	1	60	20	100	80	60	40	20
	2	50	15	80	65	50	35	20
	3	40	10	60	50	40	30	20
	4	30	5	40	35	30	25	20
III	1	28	20	20	40	20	40	20
	2	37	20	45	25	45	25	45
	3	65	25	75	50	75	50	75
	4	92	20	100	80	100	80	100
IV	1	28	20	20	40	20	40	20
	2	36	40	20	60	20	60	20
	3	44	60	20	80	20	80	20
	4	52	80	20	100	20	100	20
V	1	68	80	100	20	100	20	100
	2	76	60	100	40	100	40	100
	3	84	40	100	60	100	60	100
	4	92	20	100	80	100	80	100

*ω*_1_, *ω*_2_, *ω*_3_, *ω*_4_, *ω*_5_—angular speeds of discs, Δ*ω*—increase in angular speeds, *ω_aver_.*—average angular speed for five discs.

**Table 3:** Results of power consumption and specific comminution energy tests in the grinding of rice and corn grains for five test programs.

TP	Conf.	Corn				Rice			
		*P_i_*	*P_c_*	*E_s_*	*P_i_/P_c_*	*P_i_*	*P_c_*	*E_s_*	*P_i_/P_c_*
		kW	kW	Wh/kg	%	kW	kW	Wh/kg	%
I	1	0.93	1.09	10.78	84.95	0.93	1.09	6.30	84.91
	2	1.13	1.51	20.82	74.40	1.13	1.43	11.15	78.84
	3	1.30	1.89	29.85	68.81	1.30	1.73	14.73	75.19
	4	1.49	2.27	34.05	65.81	1.49	2.06	18.36	72.67
II	1	1.19	1.86	21.98	63.83	1.19	1.62	14.63	73.14
	2	1.09	1.70	20.57	64.06	1.09	1.42	11.53	76.70
	3	0.91	1.31	14.11	69.61	0.91	1.16	9.06	78.71
	4	0.72	0.97	9.75	73.60	0.72	0.89	6.51	80.78
III	1	0.67	0.91	10.34	72.85	0.67	0.84	6.83	79.46
	2	0.96	1.41	17.02	68.05	0.96	1.23	10.14	77.76
	3	1.53	2.52	33.53	60.60	1.53	1.99	15.97	76.85
	4	1.90	3.11	37.12	61.27	1.90	2.48	19.01	76.65
IV	1	0.67	0.91	10.34	72.85	0.67	0.84	5.62	79.46
	2	0.74	1.35	46.11	54.85	0.74	1.02	9.53	72.38
	3	0.88	1.54	92.26	56.87	0.88	1.21	11.47	72.61
	4	0.98	1.82	193.18	54.22	0.98	1.37	14.22	71.94
V	1	1.49	2.31	146.83	64.40	1.49	1.97	18.57	75.35
	2	1.54	2.65	64.69	58.04	1.54	2.08	19.99	74.03
	3	1.68	2.70	37.90	62.13	1.68	2.21	19.02	75.98
	4	1.90	3.11	37.12	61.27	1.90	2.48	19.39	76.65

**Table 4:** The coefficients of the obtained RRSB distributions for the tested configurations of disc angular velocity settings.

TP	Conf.	Corn					Rice				
		x_0_	*k*	*μ*	*σ*	Adj. *R*^2^	x_0_	*k*	*μ*	*σ*	Adj. *R*^2^
I	1	2.84	2.02	2.52	1.31	1.00	1.90	6.34	1.77	0.33	0.999
	2	1.58	2.67	1.44	0.58	1.00	1.80	4.80	1.65	0.39	0.998
	3	1.45	2.00	1.29	0.51	1.00	1.67	3.50	1.50	0.48	0.997
	4	1.50	2.81	1.34	0.52	1.00	1.52	3.05	1.36	0.49	0.998
II	1	1.87	2.80	1.72	0.64	1.00	1.72	3.79	1.56	0.46	0.997
	2	1.78	2.74	1.59	0.63	1.00	1.82	4.94	1.76	0.39	0.999
	3	1.86	2.45	1.65	0.72	1.00	1.86	5.54	1.94	0.36	0.999
	4	1.84	2.39	1.63	0.73	1.00	1.92	6.87	1.80	0.31	1.000
III	1	1.96	2.43	1.74	0.76	1.00	1.89	5.84	1.75	0.35	0.999
	2	2.34	2.55	1.63	0.68	1.00	1.85	5.11	1.70	0.38	0.998
	3	1.76	2.77	1.57	0.61	1.00	1.62	3.22	1.46	0.50	0.997
	4	1.37	2.96	1.40	0.51	1.00	1.40	2.76	1.25	0.49	0.999
IV	1	1.96	2.43	1.74	0.76	1.00	1.89	5.84	1.75	0.35	0.999
	2	1.78	2.48	1.58	0.68	1.00	1.80	4.88	1.65	0.39	0.999
	3	1.58	3.05	1.41	0.51	1.00	1.64	3.60	1.48	0.46	0.998
	4	1.65	3.00	1.48	0.54	1.00	1.54	2.13	2.78	0.47	0.998
V	1	1.62	2.85	1.45	0.55	1.00	1.57	3.51	1.41	0.45	0.999
	2	1.49	3.06	1.33	0.48	1.00	1.55	3.12	1.38	0.49	0.998
	3	1.52	3.06	1.36	0.48	1.00	1.46	2.80	1.30	0.50	0.997
	4	1.37	2.96	1.40	0.51	1.00	1.40	2.76	1.25	0.49	0.999

**Table 5:** The results of the Spearman correlation analysis between the average angular velocity of the working unit, power consumption and specific comminution energy and the parameters of RRSB distributions.

		*x*_0_	*k*	*μ*	*σ*
Corn	*ω_aver._*	−0.776 *	0.779 *	−0.714 *	−0.797 *
	*P_c_*	−0.815 *	0.733 *	−0.779 *	−0.829 *
	*E_s_*	−0.645 *	0.795 *	−0.673 *	−0.768 *
Rice	*ω_aver._*	−0.919 *	−0.877 *	−0.775 *	0.899 *
	*P_c_*	−0.890 *	−0.843 *	−0.831 *	0.902 *
	*E_s_*	−0.919 *	−0.880 *	−0.824 *	0.907 *

*: Correlation is significant at the 0.05 level, 2-tailed test of significance was used.

## Data Availability

The data were included in the manuscript and Supplementary Materials for this article. Any additional data are available on the request from the corresponding author.

## Supplementary Materials

The following supporting information can be downloaded at: https://www.mdpi.com/article/10.3390/ma15176067/s1, Table S1: Results of power consumption of each disc for an idle run of the mill for five test programs; Table S2: Results of power consumption of each disc during corn comminution for five test programs; Table S3. Results of power consumption of each disc during rice comminution for five test programs.

## Appendix A

Figure A1, Figure A2 and Figure A3 show the relationship between the average angular velocity, power consumption, and specific comminution energy and the expected value and variance of the Rosin–Rammler–Sperling–Bennet particle size distributions.
