# Supplementary material for: Energy-Dependent Particle Size Distribution Models for Multi-Disc Mill
Source: Materials (Basel). 2022 Sep 1;15(17):6067. doi: 10.3390/ma15176067 (PMC9457795; doi:10.3390/ma15176067)
Supplement: Supplementary file 1 [file materials-15-06067-s001.zip › Suplementary files.pdf]

Table S1. Results of power consumption of each disc for idle run of mill for five test programs

TP	Conf.	P_{i1}	P_{i2}	P_{i3}	P_{i4}	P_{i5}
		kW	kW	kW	kW	kW
I	1	0.18	0.10	0.12	0.16	0.37
	2	0.17	0.12	0.15	0.21	0.48
	3	0.16	0.13	0.17	0.26	0.59
	4	0.16	0.14	0.20	0.30	0.69
II	1	0.49	0.21	0.18	0.15	0.14
	2	0.46	0.19	0.16	0.14	0.15
	3	0.35	0.15	0.14	0.12	0.15
	4	0.24	0.12	0.11	0.10	0.15
III	1	0.13	0.14	0.09	0.15	0.15
	2	0.27	0.10	0.15	0.11	0.33
	3	0.43	0.16	0.23	0.20	0.50
	4	0.44	0.22	0.26	0.31	0.67
IV	1	0.13	0.14	0.09	0.15	0.15
	2	0.12	0.19	0.08	0.21	0.13
	3	0.13	0.24	0.08	0.28	0.14
	4	0.13	0.26	0.09	0.36	0.15
V	1	0.44	0.08	0.25	0.09	0.63
	2	0.43	0.13	0.26	0.16	0.55
	3	0.42	0.18	0.28	0.24	0.56
	4	0.44	0.22	0.26	0.31	0.67

TP – test program, conf. – configuration, $P_{i1, 2, 3, 4, 5}$ – idle power consumption for disc 1, 2, 3, 4 and 5 respectively

Table S2. Results of power consumption of each disc during corn comminution for five test programs

TP	Conf.	P_{c1}	P_{c2}	P_{c3}	P_{c4}	P_{c5}
		kW	kW	kW	kW	kW
I	1	0.22	0.15	0.20	0.24	0.27
	2	0.21	0.20	0.33	0.38	0.40
	3	0.21	0.25	0.42	0.49	0.52
	4	0.22	0.30	0.52	0.60	0.63
II	1	0.71	0.44	0.35	0.23	0.13
	2	0.69	0.36	0.31	0.21	0.13
	3	0.48	0.27	0.24	0.19	0.13
	4	0.32	0.20	0.17	0.15	0.13
III	1	0.19	0.24	0.14	0.22	0.13
	2	0.40	0.17	0.29	0.22	0.33
	3	0.68	0.36	0.56	0.39	0.54
	4	0.68	0.52	0.63	0.58	0.69
IV	1	0.19	0.24	0.14	0.22	0.13
	2	0.19	0.37	0.32	0.34	0.13
	3	0.21	0.50	0.37	0.34	0.13
	4	0.27	0.54	0.36	0.52	0.13
V	1	0.64	0.44	0.46	0.13	0.64
	2	0.67	0.30	0.66	0.35	0.67
	3	0.65	0.40	0.61	0.41	0.63
	4	0.68	0.52	0.63	0.58	0.69

TP – test program, conf. – configuration, $P_{c1, 2, 3, 4, 5}$ – total power consumption for disc 1, 2, 3, 4 and 5 respectively

Table S3. Results of power consumption of each disc during rice comminution for five test programs

TP	Conf.	P_{c1}	P_{c2}	P_{c3}	P_{c4}	P_{c5}
		kW	kW	kW	kW	kW
I	1	0.21	0.15	0.15	0.17	0.40
	2	0.23	0.21	0.23	0.25	0.51
	3	0.24	0.24	0.29	0.33	0.62
	4	0.26	0.28	0.35	0.41	0.76
II	1	0.67	0.36	0.25	0.18	0.16
	2	0.61	0.28	0.21	0.15	0.16
	3	0.46	0.23	0.17	0.13	0.16
	4	0.30	0.17	0.14	0.11	0.16
III	1	0.18	0.21	0.11	0.18	0.16
	2	0.37	0.14	0.21	0.12	0.38
	3	0.58	0.25	0.35	0.24	0.57
	4	0.58	0.37	0.42	0.39	0.72
IV	1	0.18	0.21	0.11	0.18	0.16
	2	0.17	0.31	0.12	0.26	0.16
	3	0.17	0.41	0.12	0.34	0.16
	4	0.17	0.46	0.13	0.44	0.16
V	1	0.59	0.12	0.41	0.12	0.73
	2	0.56	0.20	0.40	0.21	0.70
	3	0.54	0.29	0.40	0.28	0.69
	4	0.58	0.37	0.42	0.39	0.72

TP – test program, conf. – configuration, $P_{c1, 2, 3, 4, 5}$ – total power consumption for disc 1, 2, 3, 4 and 5 respectively
